# Endophytic Entomopathogenic Fungi: Their Role in Enhancing Plant Resistance, Managing Insect Pests, and Synergy with Management Routines

**DOI:** 10.3390/jof10120865

**Published:** 2024-12-13

**Authors:** Krishnamoorthy Aravinthraju, Mookiah Shanthi, Marimuthu Murugan, Ramasamy Srinivasan, Lourena Arone Maxwell, Narayanan Manikanda Boopathi, Rangasamy Anandham

**Affiliations:** 1 Department of Agricultural Entomology, Tamil Nadu Agricultural University, Coimbatore 641003, India; aravinth4697@gmail.com; 2 Safe and Sustainable Value Chains, World Vegetable Center, Tainan 74151, Taiwan; lourena.maxwell@worldveg.org; 3 Centre for Plant Protection Studies, Tamil Nadu Agricultural University, Coimbatore 641003, India; mshanthiento@tnau.ac.in; 4 Department of Biotechnology, Tamil Nadu Agricultural University, Coimbatore 641003, India; nmboopathi@tnau.ac.in; 5 Department of Agricultural Microbiology, Tamil Nadu Agricultural University, Coimbatore 641003, India; anandham@tnau.ac.in

**Keywords:** insect pathogen, plant-inhabiting, metabolites, volatiles, microbiome, compatibility

## Abstract

The interaction between plants and microorganisms plays a major role in plant growth promotion and disease management. While most microorganisms directly influence plant health, some indirectly support growth through pest and disease suppression. Endophytic entomopathogenic fungi are diverse, easily localized, and have long-lasting effects on insect pests. When inhabiting plants, these fungi alter secondary metabolites, volatile organic compounds, and microbiomes, enhancing plant resistance to pests and diseases and sometimes improving growth. However, their persistence in plant systems may be challenged by the plant’s defense mechanisms or by human interventions such as insecticides, fungicides, herbicides, and phyto-insecticides, which are common in agriculture. As effective biocontrol agents, endophytic entomopathogenic fungi can also be integrated with other pest management strategies like predators, parasitoids, and chemicals. This review will explore the impact of endophytic entomopathogens on plant systems and their compatibility with other management practices.

## 1. Introduction

The relationship between plants and endophytic microorganisms has emerged as a key insight in enhancing our understanding of plant growth and resilience. Historically, the exploration of endophytic microorganisms primarily focused on plants native to temperate regions. However, in recent years, studies have shifted their focus to tropical plants. Endophytic microorganisms are believed to affect plant growth, including stimulating growth and protecting the plant from pests and diseases. Plants are estimated to harbor one or more endophytic fungi and bacteria. Still, several studies are to be conducted on these endophytes and plants to explore their need in pest control [1], as recent studies have shown that endophytic microbes reduce pest attacks and enhance plant growth [2].

Numerous reports have been published about the interaction of endophyte microbes with plants, including mycorrhizal fungi [3]. Plant growth promoting regulators [4,5] and other fungal endophytes in grass [6,7,8]. The main endophytic organisms are fungi and bacteria [9,10]; a few algae and oomycetes are also reported as endophytic [11].

In general, endophytes are defined by many [12], but in simple terms, those organisms do not produce harmful symptoms in plant tissues [13]. Endophytes fall into two groups: those that do not develop external structures from the host and those capable of creating such structures as nodules of N_2_-fixing bacteria. Their main purpose is to protect their host plants from herbivores such as cattle and pest insects and increase plant resistance to pathogens that produce antimicrobial agents and plant growth hormones; they can also counteract adverse biotic and abiotic conditions [1].

Mainly reported fungal endophytes are Penicillium, Alternaria, Colletotrichum, Fusarium, Aspergillus, Phoma, Phomopsis, Pestalotiopsis, Xylaria, Phomopsis, Diaporthe, Acremonium, Chaetomium, Trichoderma, Curvularia, etc., each with different roles [14]. Likewise, bacterial endophytes including, Rhizobium, Bradyrhizobium, Rhodococcus, Bacillus, etc. [15,16]. Additionally, Pythium oligandrum, an oomycete algae, is reported as a growth-promoting and plant-protection endophyte [17].

Most of these endophytes are responsible for plant diseases (adverse effects), plant growth, and protection (positive effects). Endophytic pathogens, like Alternaria and Cladosporium, cause plant leaf spot diseases, which are good examples. Also, some endophytes will arrest the growth and development of endophytic pathogens and promote plant growth and pest resistance [18]. In that way, entomopathogenic endophytes play a significant role in plant protection from pests.

Even though bacteria and fungi species are reported as plant endophytes, fungi are diverse. More than hundreds of endophytic fungi were reported, with an excellent example of 257 ITS fungal endophytic genotypes from coffee plantations in Colombia, Hawaii, Puerto Rico, and Mexico [19]. Also, fungal endophytes are localized [20], establishing a long-term systemic infection for insects [13]. Hence, this review discusses the influence and compatibility of entomopathogenic fungi with their host plants and insects.

## 2. Entomopathogenic Fungi as Endophytes

Entomopathogenic fungi can infect arthropod pests in a wide species range. Almost all terrestrial ecosystems and habitats play various ecological roles [21]. In addition to soil and phylloplane, they have also been observed as endophytes and rhizosphere-competent microorganisms [22,23,24,25].

Entomopathogenic fungi are essential to biological pest control since they are the natural enemies of many insect pests [26,27]. These fungi can develop within insects after penetrating their exoskeleton or outer coating, eventually causing the host to die. Since entomopathogenic fungi are unique to insects, using them in pest management [28,29] is considered more environmentally benign than chemical insecticides [25]. This strategy is a component of integrated pest management plans, which seek to support sustainable agriculture and lessen dependency on artificial pesticides [30].

After discovering that these fungi can be endophytes with a systemic biological control function [25,31], there is concern about previously unconsidered interactions between entomopathogenic fungi and beneficial organisms, including other biological control agents. Also, the defense mechanism of insects against entomopathogenic fungi, like the exoskeleton barrier, can be eliminated. Additionally, due to feeding the endophyte colonized plants, the spores may affect the insect gut directly, increasing the high infectivity change [31]. There has been considerable use of entomopathogenic fungi for controlling many crop pests, including stem borer (*Chilo partellus*) in maize and sorghum [32], cotton leaf roller (*Sylepta derogata*) [33], potato beetle (*Leptinotarsa decemlineata*) [34], aphids (*Aphis* spp.), tea mosquito bug (*Helopeltis* spp.) that affect guava, moringa, and cashew [35,36], and wheat mites (*Amblyomma maculatum* and *A. americanum*) [37,38].

There is a growing interest in endophytic fungi due to their ability to enhance plant growth, improve nutrition, and control pests and plant diseases [39,40,41,42]. Endophytes can also increase crop yields, remove contaminants, inhibit pests, and generate fixed nitrogen or novel substances [43]. In 1866, de Bary described fungi as endophytes, but they were considered neutral, causing no harm or benefit to their plant hosts [44]. Studies focusing on endophytes have increased significantly over the last 30 years. Endophytic fungi have only recently been discovered to play an essential role in protecting plants from herbivores such as insects. In addition to providing nutrition to the host, they also enhance the plant’s ability to withstand drought, cold, and pathogens.

Endophytic microorganisms have been found to occur in every plant studied to date. Our planet is estimated to have 1.5 million different fungal species, but only a small percentage have been described [45]. Since most fungal species are significant from the perspective of environmental and biotechnological research, endophytic fungi have only been isolated from very few of the 300,000 existing plant species, and endophytes have the potential to produce new antibiotics, enzymes, dyes, and other valuable compounds. Furthermore, they can be beneficial in controlling pests and diseases and enhancing plant growth vigor by supplying nutrients or hormones to the host. Different aspects of fungal endophytes have been discussed in these aspects in several reviews [40,46,47,48,49,50].

There are various examples of entomopathogenic endophytic fungi isolated from different host plants in the literature reviews [24,46]. The results of some of the experiments conducted by a group in Brazil also showed the presence of entomopathogenic endophytic microorganisms. There were several fungi that were commonly isolated from several studied plant hosts, such as *Beauveria*, *Cladosporium*, *Cordyceps*, *Paecilomyces* (*Isaria*), and *Verticillium* (*Lecanicillium*), *Pochonia chlamydosporium* [29,51,52]. Other well-known entomopathogenic fungi include, *Metarhizium* [24,46,51], *Isaria fumosorosea*, *Beauveria brongniartii* [53], and *Akanthomyces muscarius* [54].

**Figure 1:**
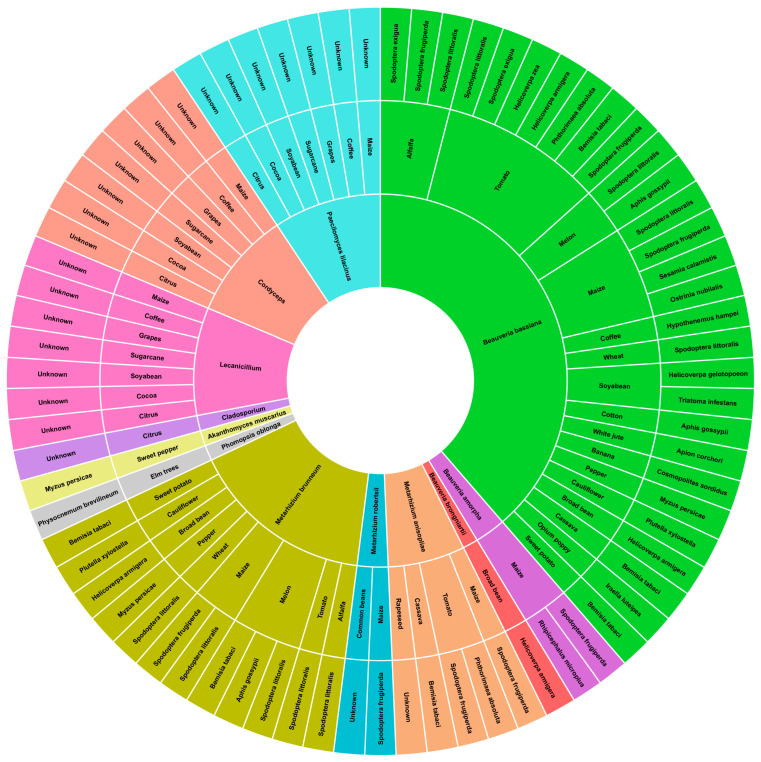
Endophytic entomopathogenic fungi and their associated host against insect pests.

The endophytic entomopathogenic fungi of this type have been identified among plants of agricultural significance (Figure 1), including *Zea mays* [55], *Glycine max* [55], *Theobroma cacao* [56], *Saccharum* [57], *Vitis labrusca* [58], *Coffea Arabica* [59], and some Citrus species [60], *Medicago sativa*, *Solanum lycopersicum*, *Cucumis melo*, *Triticum* [61,62], *Gossypium* spp. [63], *Corchorus capsularis* [64], *Musa* spp. [65], *Capsicum* spp. [66], *Brassica oleracea* [50], *Vicia faba* [67], *Ipomoea batatas* [68], *Manihot esculenta* [69], *Papaver somniferum* [70], *Brassica napus* [71], *Ulmus* spp., *Vitis vinifera*, and *Saccharum officinarum* [1].

## 3. Metabolic Changes in Host Plants Due to Endophytic Entomopathogenic Fungi

**Figure 2:**
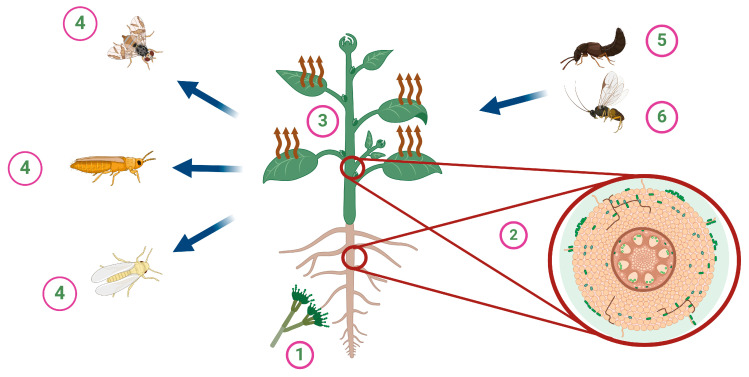
Metabolic changes in host plants due to endophytic entomopathogenic fungi. The endophytic entomopathogenic fungus (1) enters the plant system through the roots and colonizes within the plant tissues (2). This colonization may alter plant volatiles (3), secondary metabolites, and hormones, which can result in repelling insect pests (4) and attracting natural enemies, such as predators (5) and parasitoids (6).

As mentioned above, many entomopathogenic fungi are colonizing different host plants. However, these entomopathogenic fungi may alter the plant nutrients and/or defensive compounds within the plant, which may adversely impact the plant’s ability to serve as a food source for herbivores or repel them (Figure 2) [72,73,74,75,76,77]. Those adverse effects may be due to plants’ production of phenolics, terpenes, flavonoids, alkaloids, and volatile organic compounds (VOCs) as secondary metabolites [78,79]. Also, some hormones will be produced in plants that may induce resistance against herbivores and plant growth.

### 3.1. Changes in Volatile Blends

In different metabolic changes, VOCs play a main role as preventive measures and decide the attraction of natural enemies towards plants and the repulsion of herbivores away from plants. In plants, microbes play a significant role in producing and releasing specific plant volatiles [80,81]. Insect population dynamics may be altered at higher trophic levels through endophytic colonization by entomopathogenic fungi [3,5,6,82]. As a result of herbivores influencing plant volatiles, changes in volatiles and attraction to natural enemies have been observed in response to endophytic microorganisms [4,83,84].

The *Beauvaria* colonization alters the plant’s chemical volatiles, some of which are reported to attract natural enemies, and some possess antimicrobial properties. Furthermore, a study reported the effects of endophytic *B. bassiana* (Bals.) Vuill and *Metarhizum brunneum* (Petch) (Ascomycota: Hypocreales) on volatile compounds released by the leaves from melon plants [85]. Subsequent aphid infestation results in quantitative and qualitative differences in the leaves that *Beauvaria* and *Metarhizium* colonized with and without aphid infestation. This may be caused by the defense responses triggered by endophytic fungi colonized plants, which in turn cause changes in volatile blends [86]. Defense compounds such as cis-jasmone and methyl jasmonate were increased simultaneously with the shift in volatile emission [87,88].

The melon plants that *B. bassiana* colonized and infested with *Myzus periscae* produced a more volatile compound, 6-methyl-octadecane, which may serve as an attractant to natural enemies or repellent to pests [89], but further studies will be required.

Aphid immune responses were reduced by endophytic colonization by *B. bassiana* (altering aphid symbionts), causing aphids to become more attractive to predators when consuming endophytically colonized plants [73,74,75,77,90]. There was also a significant amount of VOC compound 1-iodo-2-methyl-undecane in colonized non-aphid-infested melon plants, previously reported as a floral volatile [91].

In wild cotton, plants colonized by *B. bassiana* release natural enemy attractants such as 4-methyl-octane, a-caryophyllene, and (E)-hex-2-en-1-ol [92]. Caryophyllene and alpha-caryophyllene were released more significantly in the presence of endophytic colonization. However, this effect was neutralized after caterpillar feeding [93,94].

### 3.2. Influence on Secondary Metabolites

Next to volatile compounds, plant secondary metabolites play a significant role in plant protection against pests. These alterations in secondary metabolites are mainly due to herbivore feeding, but endophytic colonization also plays a vital role.

*O. nubilalis* feeding on maize with *B. bassiana* as an endophyte displayed fewer insects with mycoses [1]. It was suggested that, since no conidia were found inside the host plant, the mechanism of action may involve fungal metabolites, which are responsible for insect deterrents or antibiosis [95].

Different researchers have found some defensive compounds like lipo-chitooligosaccharides (LCO) [96], root exudates like sugars, amino acids, organic acids, phenolic compounds [97,98], strigolactone (SL) [99], and arabinogalactan proteins (AGPs) [100], are reported as a result of colonization of entomopathogenic fungi. Sometimes, these secondary metabolites act as barriers to endophytic fungus colonization. In those cases, endophytic fungi create detoxifying and degradation enzymes, such as cellulases, chitinases, amylases, β-1,3-glucanases, and lactases, to overcome the harmful effect of plant metabolites. Furthermore, fungal metabolites are crucial to various interactions between the fungus, plants, and its insect host. They assist in reducing abiotic and biotic stress or mediate intra or inter-specific communication functions in plants [101]. The synthesis of root exudates by host plants has increased the presence of *Metarhizium* in some plants [102].

In the case of melon, the first report of 2-ethylhexyl nonyl sulfite was identified as a biomarker in endophytically colonized plants when infested with cotton aphids [63].

### 3.3. Influence on Plant Hormones and Enzymes

Plant hormones and enzymes have less of an effect on plant protection but have a significant role in plant growth. Combining entomopathogenic fungi biocontrol with plant growth promotion in agriculture will have a more substantial impact [31]. Endophytes produce a variety of phytohormones, such as indole-3-acetic acid (IAA), cytokines, and other plant growth-promoting substances, which contribute to this effect [103]. Endophytes also enhance the uptake of nutritional elements by their hosts by increasing nitrogen uptake [104] and phosphorus uptake [105].

Endophytic *Beauveria* strains produce a variety of hydrolytic enzymes, like proteases and chitinases that can harm ticks, suggesting that these enzymes play an important role in pathogenesis. The endophytic strains also formed appressoria during tick cuticle penetration [106].

### 3.4. Enhanced Antimicrobial Compounds

Even though microbes alter the plant metabolites and VOCs, their colonization will result in antimicrobial compounds in plants as they are also considered foreign compounds to the plant system and are harmful to those endophytes and other plant disease-causing microbes. Some antifungal or antimicrobial compounds may be enhanced due to colonization by entomopathogenic fungi. Antimicrobial properties are known to be associated with some compounds such as benzaldehyde and (2Z,13E)-octadeca-2,13-dien-1-ol, which are found in wild cotton plants that are colonized by endophytes and are infested with pests [107,108,109,110,111,112,113,114,115]. Benzaldehyde exhibits some antimicrobial activity against *Lecanicillium lecanii* and impacts the growth of the fungus, the production of conidia, and the germination of spores [116]. In melon, defense compounds such as 1-iodo-2-methylundecane and oxolan-2-one were present in large quantities when the fungus colonized the melon plants, exhibiting a defense mechanism against it. Additionally, in melon plants, hexadecane-4-yl 2,2,2-trifluoroacetate exhibits antimicrobial activity against bacteria and fungi [117,118,119], whereas 6-methyl-octadecane showed anti-nematode activity [120]. Similarly, 1-iodo-2-methylundecane and 6-methyl-octadecane have combined action against *Paecilomyces lilacinus*, an entomopathogenic fungus [121]. However, endophytic organisms can detoxify those antimicrobial compounds in several cases.

## 4. Endophytic Entomopathogenic Fungi Discovered So Far

The direct and adverse indirect effects on plants and endophytic EPFs are becoming successful in controlling herbivores. According to Paulo Teixeira Lacava (2014), endophytic microorganisms play a significant role in controlling insects since they act as a protective factor against insects [1]. Various genera of entomopathogenic fungi can colonize a wide range of host plants as endophytes, which provides an exciting opportunity to improve their effectiveness [25]. An endophytic fungus, *Phomopsis oblonga*, was found to protect elm trees from the beetle *Physocnemum brevillineum*, which spreads the Dutch Elm disease through the transmission of the pathogenic fungus *Ceratocystis ulmi*. A review provided many examples of fungal endophytes that control insects and their ability to produce toxins that protect plants against herbivores [40]. The first report on this correlation demonstrated that *Epichloe typhina* produced a toxin in *Festuca arundinacea*, its host plant [122]. Inoculation of granular and aqueous formulations of *B. bassiana* controlled the infestation of European Corn borer, *Ostrinia nubilalis*, in *Zea mays* (Maize) [1].

The strains *B. amorpha* [123,124] have been employed against a major maize insect pest (*Spodoptera frugiperda*), and the results have shown that endophytes from maize are as effective as commercial entomopathogenic strains in Brazil for the control of *S. frugiperda*. The same strains have also been tested in vivo and in vitro against an ectoparasite bovine tick, *Rhipicephalus microplus*, and the strains reduced the egg weight and reproduction efficiency of females. Field tests indicated that the endophytic strain was the most effective, followed by a *B. bassiana* strain obtained from insects [106]. Until now, this was the first field study in Brazil using endophytic entomopathogen fungi to combat ticks, and the results showed an increase in mortality of 32% compared to controls. The mortality of African ticks using insect isolates entomopathogens can be as high as 85 percent [1], indicating that a search for new endophytes in combination with improved delivery conidia can lead to an increase in mortality, leading to the possibility of replacing synthetic compounds by biological control techniques. *B. bassiana* (Balsamo) Vuillemin (Ascomycota: Hypocreales) grows directly through the cuticle of its host, growing into the body of the insect, increasing throughout the body, and eventually killing it [32].

The stem gall wasp *Iraella luteipes* (Thompson) (Hymenoptera: Cynipidae) larvae that were discovered dead inside opium poppy stems were naturally infected with *B. bassiana* strain EABb 04/01-Tip [70]. When treated artificially into the opium poppy seeds, vertical transfer of an entomopathogenic fungus from mother plants that have been endophytically colonized [125] provided resistance against *I*. *luteipes*.

The endophytic *B. bassiana* isolate has suppressed the stemborer (*Sesamia calamistis*) in maize [95]. Endophytic *B. bassiana* strains significantly reduced the survival and damage caused by banana stem weevils (*Cosmopolites sordidus*) in tissue-cultured banana plants [65]. Endophytic *B. bassiana* is also reported to control coffee berry borer (*Hypothenemus hampei*) [46]. Furthermore, *B. bassiana* endophytic colonization on white jute decreased stem weevil infestation [64].

Few studies have demonstrated the endophytic effect of *B. bassiana* and *M. brunneum* on alfalfa, melon, tomato, and wheat [61,62] and *B. bassiana* on tomato [73] against *S. littoralis*. Also, 12 isolates of *B. bassiana* and one isolate each of *M. anisopliae* and *M. robertsii* were shown to colonize maize plants, with the highest rate being demonstrated by a *B. bassiana* isolate (LPSc 1098) during foliar spraying. As a result, *S. frugiperda* in maize has decreased larval growth, pupal survival, developmental stages, and a shorter lifespan. Additionally, the colonization decreased reproductive success, longevity, and fecundity among females [50].

The impact of endophytic colonization by the entomopathogenic fungi *B. bassiana* ARSEF 3097 and *Akanthomyces muscarius* ARSEF 5128 on sweet pepper plant infestations has altered the life cycle and behavioral response of the *Myzus persicae* var. *nicotianae* [65].

Maize seeds coated with microsclerotia of *Metarhizium* spp. induced the growth traits of plants and reduced the survival of *S. frugiperda* [126]. The potential of four endophytic fungal species, namely, *Beauveria* spp., *Aspergillus* spp., *Curvularia* spp., and *Chaetomium* spp., against *S. frugiperda* in maize was tested [127]. Studies showed that, at 12 days after treatment, *Beauveria* spp. (isolates at 1 × 10^6^ conidia/mL) recorded high mortality under laboratory conditions.

## 5. Compatibilities with Other Management Practices

**Figure 3:**
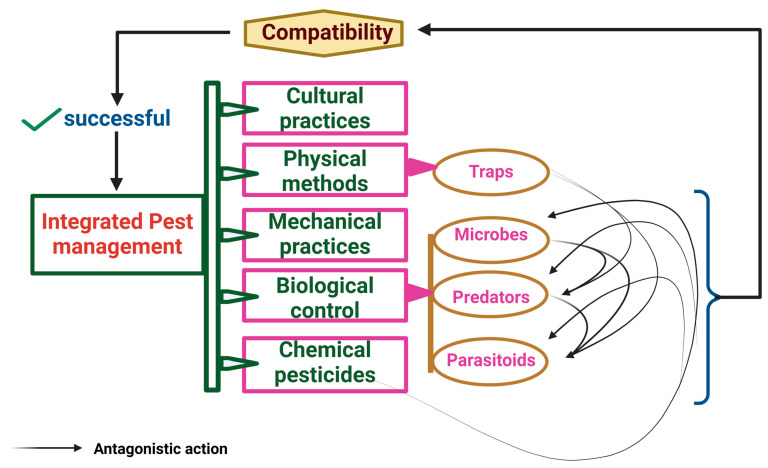
Compatibility of IPM components for its success. Microbes have a two-way interaction: they can be antagonistic to natural enemies while also being affected by chemical pesticides. Therefore, their compatibility with other integrated pest management (IPM) practices is crucial for successful pest management.

Problems with any reasonable solutions are always expected. Likewise, here, for all successful endophytic fungal organisms, the artificial problem is one of the management practices we follow, like pesticides and botanicals. On the other hand, the endophytic organism should not cause problems to the entomophagous natural enemies. However, employing entomopathogenic fungi in integrated pest management (IPM) in combination with other biocontrol agents, such as parasitoids and predators [66,128,129], and with botanicals will lead to environmental safety measures [68] (Figure 3). Similarly, compatibility between all the management measures is necessary.

### 5.1. Compatibility of Endophytic Fungi with Entomophagous Arthropods

By combining entomopathogenic fungi with entomophagous arthropods, the contact action of these fungi and the ability of predators and parasitoids to search for their prey can be utilized to ensure that fewer pests evade treatment [130,131]. A combination of different biological control agents may be additive in suppressing pest populations [132]. Several studies have combined entomopathogenic fungi with other biological control components, including predators, parasitoids, and nematodes, to evaluate their safety and effectiveness [133,134,135,136,137].

*Chyrysoperla carnea* larvae prefer to feed on aphids reared on plants colonized with *B. bassiana* than those grown on control plants, in a choice bioassay [129]. It was also found that the number of aphids parasitized by *Aphidius colemani* and their sex ratio was not influenced by whether the aphids had fed on *B. bassiana* colonized plants. Therefore, Endophytic entomopathogenic fungi can be part of integrated pest management programs with predators and parasitoids. Occasionally, the interval between the release of natural enemies such as predators and parasitoids and the application of fungi affects the number of aphids consumed and attacked by these predators and parasites [138,139,140].

In some studies, parasitoids colonized by entomopathogenic fungi endophytically were reported to induce analogous effects [66,67,128]. However, the impact of colonization on predators remains unknown and needs further investigation.

### 5.2. Compatibility of Entomopathogenic Fungi with Pesticides

Several studies have been conducted to assess the effects of pesticides on entomopathogenic fungi [141,142]. Entomopathogenic fungi can respond synergistically, antagonistically, or neutrally to insecticides [143]. Pesticides incompatible with these entomopathogens can inhibit their development and reproduction, affecting IPM [141]. Data concerning compatibility are essential before such associations (chemical insecticides and entomopathogens) are used in the field [142].

Combining selective insecticides with entomopathogens allows greater control efficiency, reducing insecticide application, reducing environmental contamination hazards, and preventing pest resistance [144,145]. Through their effects on growth, sporulation, and germination, insecticide application with entomopathogenic fungi may impact the latter’s effectiveness. Therefore, testing an insecticide’s compatibility with entomopathogenic fungi is crucial. Conidial survival may be affected by interactions with environmental variables, agrochemicals, biopesticides, and/or chemical plant protection products [141]. Thus, the compatibility of entomopathogenic fungi with different pesticide groups is discussed.

#### 5.2.1. Insecticides

Entomopathogenic fungi and insecticides at sublethal concentrations work synergistically to enhance insect mortality [146]. This will also lessen environmental contamination, the chance of resistance developing, and the dosage of highly beneficial insecticides. In contrast, some in vitro research shows that pesticides suppress the growth and development of *B. bassiana* [141].

*M. anisopliae* and *B. bassiana* were shown to be most susceptible to dicofol (0.07%), cypermethrin (0.009%), deltamethrin (0.005%), and chlorpyriphos (0.05%) [147]. However, *M. anisopliae* was compatible with spinosad and indoxacarb [148]. In some strains of *B. bassiana*, indoxacarb significantly inhibited sporulation and spore viability but did not affect the species’ radial growth [149]. Chlorantraniliprole 18.5% SC was observed to be compatible with *B. bassiana* based on their experimental results [150]. In contrast, *M. anisopliae* at various concentrations (0.6, 0.3, 0.15, and 0.075%) showed a deleterious effect on spore germination, but 76 percent spore germination was only found at the lowest dosage of 0.037%. In the same trial, other insecticides that were shown to be compatible with *B. bassiana* and *M. anisopliae* were Lambda-cyhalothrin 4.9% CS, Novaluron 10% EC, Emamectin benzoate 5% WG, and Indoxacarb 15.8% EC.

The impact of five pesticides on the vegetative growth of *M. anisopliae* was investigated [151]. Findings indicated that imidacloprid had the lowest inhibitory effect (11.1%), with deltamethrin (36.7%), cypermethrin (36.7%), thiodicarb (53.5%), and chlorpyrifos (69.2%) following closely behind. A study showed imidacloprid’s compatibility with *B. bassiana* and *M. anisopliae*, demonstrating the two entomopathogens’ high conidial production and spore germination % [152]. However, deltamethrin exhibited the greatest vegetative growth on both. When researchers examined three different imidacloprid doses (0.5 × Dose of Field, 1 × Dose of Field, and 2 × Dose of Field) against *B. bassiana*, the lowest field dose had a negligible inhibitory effect (5%) [141,153]. found that imidacloprid exhibited the least growth inhibition for *M. anisopliae* at lower concentrations.

Thiodicarb’s effects on the vegetative growth of *M. anisopliae* were observed [143]. High levels of inhibition (>60%) were seen in their results. Comparatively, it was demonstrated that imidacloprid was more effective than cypermethrin in increasing *M. anisopliae* conidial production [146]. It was also discovered that *M. anisopliae* could tolerate the lowest imidacloprid dosage [154]. Since *B. bassiana* and *M. anisopliae* could metabolize and release chemicals as secondary nutrients, imidacloprid, which is neurotoxic to insects, did not negatively affect them.

Apart from these reports, more compatible entomopathogenic fungi with newer insecticides should still be explored. From this, we can develop and promote better-integrated pest management packages for different pests.

#### 5.2.2. Fungicides

Fungicides will probably have the most significant effect on entomopathogenic fungi of all pesticides [155]. The compounds, particularly fungicides utilized to combat plant pathogens, may also have adverse effects on entomopathogenic fungal populations, which could decrease their ability to regulate pest populations [23,156,157]. However, because of the severe incompatibility between fungicides and entomopathogenic fungi, several strains of fungicide-resistant fungi were created through genetic modification [158].

Of the four fungicides tested, only Mancozeb 75% WP showed some degree of safety when tested on *B. bassiana* and *M. anisoplise* with average spore germination at lower concentrations (0.5 and 0.25%) [150]. In contrast, Carbendazim 50% WP, Hexaconazole 5% EC, and Propiconazole 25% EC completely inhibited their action at all concentrations. Also, there was complete growth suppression of *M. anisopliae* in propiconazole, carbendazim, and flusilazole at the recommended field dose [159]. It has also been reported that carbendazim and mancozeb are highly toxic to *Nomuraea rileyi*. Carbandazim, propiconazole, chlorothalonil, and hexaconazole were found to be highly harmful to *N. rileyi* by ultimately retarding its growth. In contrast, captan and triadimefan were relatively safe for *M. anisopliae* [160].

The effectiveness of *B. bassiana* may be negatively impacted by using some fungicides. Some fungicides work well with *B. bassiana* [161]. A compatibility study highlighted the significance of conidial germination [142]. The concurrent application of copper oxide, metalaxyl, and mancozeb with *B. bassiana* decreased insect infection, indicating that the fungicides prevented germination on the cuticle [162]. However, the control efficacy might be fine when fungicides are applied two or more days after fungal treatment. The fungicide did not significantly influence fungal infection or growth within the insect host. Mancozeb, which inhibited *B. bassiana* mycelial growth in laboratory culture, was found to have a substantial effect on the fungus in the field based on fungal-induced mortality of the Colorado potato beetle, *Leptinotarsa decimlineata*. Nonetheless, the field inhibition was lower than in the lab, indicating that mancozeb does not eradicate *B. bassiana* from the field. Metalaxyl had little effect in the field despite minimal suppression of *B. bassiana* in the lab [163].

Twelve fungicides were tested, among which metalaxyl (0.1%), thiram (0.2%), and chlorothalonil (0.1%) showed the least amount of suppression of vegetative growth and spore germination of *B. bassiana* and *M. anisopliae* [147]. Benomyl (0.1%), orthocide (0.2%), mancozeb (0.2%), tebuconazole (0.1%), pentachloronitrobenzene (0.1%), hexaconazole (0.1%), propiconazole (0.1%), difenoconazole (0.1%), and copper oxychloride (0.2%) were the other nine fungicides that were incompatible and completely or strongly inhibited vegetative growth and spore germination. Mancozeb fungicides inhibited *B. bassiana* on agar media in the lab. In contrast, mancozeb had a detrimental effect on the species abundance of potato leaves in the field [163].

Insecticide-compatible entomopathogens and compatibility with fungicides may be incorporated into integrated pest and disease management practices.

#### 5.2.3. Acaricides

Alongside insecticides and fungicides, acaricides are also essential pesticides in agriculture. However, few studies have explored the compatibility between entomopathogenic fungi and acaricides. Recent research showed that *Metarhizium* spp. conidia and microsclerotia were more compatible with synthetic acaricides [164]. Additionally, different isolates of *Metarhizium* demonstrated varying germination and growth patterns when synthetic acaricides were incorporated into artificial media [153]. Combining deltamethrin with *Metarhizium pingshaense* LCM S09 conidia resulted in greater tick control than either treatment alone, especially in ticks susceptible to this synthetic acaricide [165].

A combination of *Beauveria bassiana* and spirodiclofen produced a synergistic interaction in eggs, larvae [166], and adults [167] of *Tetranychus urticae*. However, when *B. bassiana* was combined with spiromesifen, fungal spore germination was reduced [168]. Later studies showed an additive interaction in both larvae and eggs of *T. urticae* [166].

Among the twelve acaricide formulations tested, avermectins and pyrethroids were compatible with *B. bassiana*. In contrast, acaricides from organophosphate and organostannic chemical groups significantly affected conidial germination, vegetative growth, and sporulation [169]. Similarly, a mixture of etoxazole and *B. bassiana* resulted in an antagonistic interaction, with a slight reduction in mycelial diameter [166].

#### 5.2.4. Herbicides

Herbicide sensitivity is typically higher in entomopathogenic fungi [170]. Due to frequent application, herbicides can accumulate in the soil, which could reduce the fungi’s effectiveness. Depending on the type and strain of the fungus, herbicides can have different inhibitory effects on their development and sporulation processes [171]. Certain herbicides and their soil residues can negatively affect entomopathogens, even at low concentrations, making them incompatible with these agents [170,172].

To minimize any adverse effects on biocontrol efficiency and incorporate *B. bassiana* into the integrated crop protection program, it is essential to understand the compatibility of *B. bassiana* with commonly used herbicides when choosing appropriate compounds for treatment scheduling [173]. When considering the detrimental effects of the herbicides on the various developmental stages of *B. bassiana*, it was found that amidosulfuron and dicamba had a lower fungistatic impact than the other herbicides tested, particularly on the processes of vegetative mycelial growth and sporulation [172]. An experiment by Celar and Kos (2016) reported that at 100% dosage, all herbicides in the test exhibited a robust inhibitory effect on conidial germination [174]. The inhibition of conidial germination exhibited a range of 82% with isoxaflutole and 100% with fluorochloridone, pendimethalin, and prosulfocarb. Inhibition rates raised to 96–100% at a 200% dose.

Herbicides also significantly impact the growth and development of entomopathogenic fungi, but there is less focus on this.

#### 5.2.5. Phyto-Pesticides

The effectiveness of treating whiteflies, *B. tabaci* with individual neem (azadirachtin) extracts and fungal entomopathogens (*B. bassiana* and *P. fumosoroseus*), was enhanced through topical administration [175,176]. Compared to separate treatments of *B. bassiana* and neem, the combined application produced 27.6 and 20.5% higher *B. tabaci* nymphal mortality [176]. When *P. fumosoroseus* and azadirachtin were combined, *Bemisia argentifolii* nymphal mortality could reach 90% [175].

Aqueous extracts of two medicinal herbs (*Calotropis procera* and *Inula viscosa*) along with endophytic fungal entomopathogens *B. bassiana* and *M. brunneum* were applied in combination to affect the survival and growth of the sweet potato whitefly (*Bemisia tabaci*) [68]. Endophytic *B. bassiana* combined with *C. procera* extract was found to have an additive effect on the mortality of whitefly developmental stages. Conversely, the combined impact of applying endophytic *M. brunneum* with either plant extract was more significant in each case than the effects of individual treatments, but they were sometimes additive.

Together with *B. bassiana*, *L. lecanii*, and *M. anisopliae*, two botanical pesticides, Tondexir (hot red pepper extract in mineral oil 85% EC) at 2.5 mL/liter of water and Palizin (coconut soap 65% SL) at 1.5–2 mL/liter of water were tested to control *Galleria mellonella*. Comparing the germination of *B. bassiana* to that of *L. lecanii* and *M. anisopliae*, the effects of Tondexir and Palizin were noticeably different [177].

Recently, phyto-insecticides have gained importance in biorational management. They may have an additive effect or enhance the impact of entomopathogens.

## 6. Conclusions

The study of epiphytic and endophytic entomopathogenic fungi against different insect pests should be continued to the next level to explore their impacts on plant systems. The alterations in plant metabolites, volatile organic compounds, hormones, and antimicrobial compounds will enhance the effect of those entomopathogens, both in plant growth and defense against pests. Exploring these alterations will identify pest-repellent and attractant compounds of natural enemies, induced plant antibiosis, etc., resulting in a tritropic interaction between entomopathogens, plants, and insects. However, the challenge here is the compatibility of entomopathogens with the other management practices followed. The compatibility of entomopathogens with entomophagous arthropods, like predators and parasitoids will pave the way for incorporating simultaneous integrated pest management. The compatibility of entomopathogens with pesticides will increase their persistence period inside the host plant. Hence, futuristic invertebrate pathology in an endophytic way will lead to efficient plant protection against insect pests.
